# Expression of D-type cyclins in colon cancer and in cell lines from colon carcinomas

**DOI:** 10.1038/sj.bjc.6602709

**Published:** 2005-07-12

**Authors:** A Mermelshtein, A Gerson, S Walfisch, B Delgado, G Shechter-Maor, J Delgado, A Fich, L Gheber

**Affiliations:** 1 Department of Clinical Biochemistry, Faculty of Health Sciences, Ben-Gurion University of the Negev and Soroka University Medical Center, Beer-Sheva, Israel; 2 Department of Chemistry, Faculty of Natural Sciences, Ben-Gurion University of the Negev, Beer-Sheva, Israel; 3 Colorectal Unit, Faculty of Health Sciences, Ben-Gurion University of the Negev and Soroka University Medical Center, Beer-Sheva, Israel; 4 Department of Pathology, Faculty of Health Sciences, Ben-Gurion University of the Negev and Soroka University Medical Center, Beer-Sheva, Israel; 5 Department of Gastroenterology, Faculty of Health Sciences, Ben-Gurion University of the Negev and Soroka University Medical Center, Beer-Sheva, Israel

**Keywords:** cyclin D1, D2, D3, colon cancer, HT29, LoVo39, proliferation, differentiation

## Abstract

Cyclins D1, D2 and D3 play important roles in cell proliferation and differentiation. Although their abnormal expression has been linked to cancer development and progression in a number of tissues, the expression of cyclin D2 and D3 proteins in colon cancer has not yet been characterised. In this study, we examined cyclin D1, D2 and D3 protein expression by Western blot analysis in tumour and adjacent normal colon tissues of 57 patients. In addition, we examined D-type cyclins protein expression in HT29 and LoVo39 cell lines from colon carcinomas, as a function of induced proliferation and differentiation. In both cell lines, the expression of the three D-type cyclins increased as a result of induced proliferation, whereas the expression of cyclin D3 increased as a result of induced differentiation. In colon tumours, cyclin D1 was overexpressed in 44%, cyclin D2 was overexpressed in 53% and cyclin D3 was overexpressed in 35% of the cases. We also found that in 16% of the cases, cyclin D3 protein expression was reduced in the tumour, as compared to the adjacent normal tissue. Examination of D-type cyclin protein overexpression in relation to the TNM stage of the tumours revealed that overexpression of cyclins D1 and/or D2, but not cyclin D3, is linked to colon carcinogenesis and that overexpression of cyclin D2 may be related to a higher TNM stage of the tumour.

In proliferating eukaryotic cells, the progression from G1 phase to S phase of the cell cycle is controlled by the expression of a subset of cell cycle control genes. These include the D-type cyclins genes, which consist of three family members, cyclin D1, D2 and D3. The regulatory function of the D-type cyclins is mediated by their interactions with cyclin-dependent kinases (CDK) 2, 4 and 6 (Matsushime *et al*, 1992) and the retinoblastoma susceptibility gene product, Rb. Phosphorylation of Rb by cyclin D in complexes with CDK4 or CDK6 in mid- to late G1 phase is believed to trigger the onset of S phase by inducing the release of E2F transcription factors from growth-inhibitory Rb complexes (Weinberg, 1995). The free E2F proteins are believed to transcriptionally activate genes involved in the activation and maintenance of DNA synthesis.

Given the critical role of D-type cyclins in cell cycle regulation, their abnormal or untimely expression has the potential to disrupt the cell cycle, and therefore assign them as proliferation-promoting genes or oncogenes (for reviews, see Bartkova *et al*, 1997; Lee and Yang, 2003). Indeed, overexpression of the D-type cyclins has been reported in a large number of tumours (Sicinski *et al*, 1996; Zhang, 1999; Zhang and Li, 1999; Florenes *et al*, 2000; Ebert *et al*, 2001; Ito *et al*, 2001; Wong *et al*, 2001). However, not all three D-type cyclins are overexpressed in the same tumour and the three cyclins are differentially linked to poor outcome of the disease. For example, overexpression of cyclin D2 in gastric cancer was shown to correlate with disease progression and poor prognosis (Yasogawa *et al*, 1998; Takano *et al*, 1999, 2000). On the other hand, cyclin D2 mRNA and protein were absent in breast cancer cell lines and primary cancer tissues, while in normal breast epithelial cells, cyclin D2 was abundantly expressed (Evron *et al*, 2001). Overexpression of both cyclins D1 and D3 has been observed in malignant melanomas (Florenes *et al*, 2000), pancreatic cancer (Ito *et al*, 2001) and ductal carcinoma of the breast (Wong *et al*, 2001). However, the linkage to prognosis was different in all these cancers. In breast and pancreatic carcinomas, cyclins D1 and D3 were differentially expressed, but neither was linked to prognosis. On the other hand, in melanomas, cyclin D3, but not D1, was clearly linked to poor clinical outcome (Florenes *et al*, 2000). These reports clearly indicate a complex and perhaps tissue-specific function of cyclins D1, D2 and D3 in cancer cells. Therefore, to understand their contribution to carcinogenesis, a detailed characterisation of their expression in cancers has to be provided for each tissue.

Overexpression of cyclin D1 in colon cancer is well established (Arber *et al*, 1996, 1999; Sutter *et al*, 1997; Bukholm and Nesland, 2000; Kristt *et al*, 2000; Holland *et al*, 2001; Utsunomiya *et al*, 2001). However, the linkage to prognosis is not clear (Bukholm and Nesland, 2000). Cyclin D1 expression in colon tumours is probably linked to its cell cycle-promoting activity in this tissue since expression of cyclin D1 antisense inhibited growth and tumorigenicity of human colon cancer cells (Arber *et al*, 1997). A recent report indicated that in colon polyps, cyclins D1 and D2 seem to be overexpressed as compared to normal tissue, while the expression of cyclin D3 in polyps is similar to that in normal colon tissue (Bartkova *et al*, 2001), which suggests a differential function of the three D-type cyclins in neoplastic transformation of the colon.

Since the expression of cyclins D2 and D3 in colon cancer tissue has not yet been demonstrated, in the present study we characterise the expression of the three D-type cyclins in malignant colorectal tissues and in cell lines from colon carcinomas, as a function of induced proliferation and differentiation. Our objective is to determine the role of the three D-type cyclins in colon tumorigenesis and the relation of their expression to the clinical outcome of the disease.

## MATERIALS AND METHODS

### Patients and tissues

Human colon tissues were obtained from the surgical specimens of 57 patients (37 male 20 female patients, mean age 66.5 years) who had undergone surgical resections for primary sporadic colorectal adenocarcinomas at the Soroka Medical Center. Tissue was removed under a protocol approved by the Ethical Institutional Review Board. Written informed consent was obtained from all patients. A piece of the tumour and a piece of morphologically normal colonic tissue were removed from a site that was 5 cm from the lesion and transferred to the laboratory. Samples were stored frozen at −80°C until use. A total of 10 tumours were located in the right, three in transverse, 27 in the left colon and 17 in the rectum. The tumours were classified into four stages according to the TNM cancer staging system: stage I (T_1_–T_2_, N_0_, M_0_) (*n*=9); stage II (T_3_–T_4_, N_0_, M_0_) (*n*=29); stage III (any T, N_1–2_, M_0_) (*n*=10); and stage IV (any T, any N, M_1_) (*n*=9). In addition, the tumours were classified according to their grade: G1, moderately to well differentiated (*n*=14); G2, moderately differentiated (*n*=38); G3, poorly to moderately differentiated (*n*=2); and G4, poorly differentiated (*n*=3).

### Cell lines

We examined D-type cyclin protein expression in HT29 and LoVo39 colon carcinoma cell lines. These two cell lines were chosen because of their differential expression of cyclin D2, cyclin D2 being expressed in LoVo39 cells, but not in HT29 cells (Lukas *et al*, 1995). HT29 cells were grown in DMEM medium and LoVo39 cells were grown in DMEM/F-12 (HAM)1:1 (Beit Haemeq, Israel), in equal volumes, at 37°C in a humidified atmosphere of a 5% CO_2_ incubator (Tuttnauer, USA). Both media were supplemented with 10% fetal calf serum, 2 mM L-glutamine and 15 antibiotic–antimycotic solution containing 10 U *μ*l^−1^ penicillin, 10 *μ*g *μ*l^−1^ streptomycin and 1250 U ml^−1^ nystatin (Beit Haemeq, Israel).

### Induction of proliferation and differentiation

Cells were seeded at a density of 5 × 10^4^ cells ml^−1^ and grown for 24 h prior to treatment. Cell viability was assessed by Trypan blue (Sigma, USA) dye exclusion assay. For induction of proliferation, cells were first arrested in G1 phase of the cell cycle by incubation in low-serum medium − 0.5% serum for 24 h for HT29 cells and 0.5% serum for 72 h for LoVo39 cells. Then, cells were released from arrest by replacing the low-serum medium by medium containing 10% serum. For induction of differentiation, cells from both cell lines were incubated in medium containing various concentrations of sodium butyrate (NaB; Sigma, USA) for 24 h.

### Western blotting

Primary colon tissues (∼20 *μ*l) were sonicated, homogenised in MB1 buffer (0.5 MTris/HCl pH 7.5, 0.5 M PIPES pH 6.8, 2 mM EDTA, 1 mM EGTA, glycerol, 0.176 mM NaCl and 0.02% Na azide) and centrifuged for 20 min at 4°C. The supernatant was mixed with sample buffer and boiled for 5 min. Cell line lysates were prepared by resuspending cells in 150 *μ*l of lysis buffer (50 mM HEPES pH 7.5, 150 mM NaCl, 10% glycerol, 1% Triton X-100, 1.5 mM MgCl_2_, 1 mM EGTA, 20 mM sodium pyrophosphate, 50 mM NaF, 3 mM EDTA and 2 mM sodium orthovanadate) supplemented with protease inhibitors complete cocktail (Boehringer Mannheim, Germany) and 0.2 mM DTT for 10 min on ice. Lysates were centrifuged and the supernatant was collected. Proteins (5–30 *μ*g) were fractionated on 12% SDS–polyacrylamide gel and transferred onto polyvinylidene fluoride membrane (Millipore, USA). Membranes were blocked with 5% low-fat milk in phosphate-buffered saline (PBS) and then incubated with the appropriate antibodies. The membranes were then washed five times with PBST (PBS+Tween) plus 1% BSA, and incubated with horseradish peroxidase-conjugated secondary antibody for 2 h at room temperature. After washing three times with PBST (PBS+Tween) plus 1% BSA, the protein bands were detected by Western lighting plus chemiluminescence reagent (NEN, USA). A semiquantitative approach was used to determine the degree of change in expression of cyclin D-type proteins. For this purpose, Western blot films were scanned and digitised. The intensity of a rectangular area that included a band of interest was determined by ImageJ software. We then carried out several normalisations steps. The background of each band was subtracted by measuring the intensity of the same rectangular area near the band of interest. To normalise the total protein quantity in each sample, the band intensity of cyclin D-type proteins was divided by the band intensity of *β*-actin in the same sample. In proliferation-induction experiments, cyclin D protein expression following release from G1 arrest was normalised to the expression at the *t*=0 time point. In the differentiation-induction experiments, cyclin D protein expression in NaB-treated samples was normalised to the control. For each case of colon cancer patient, cyclin D protein expression in the tumour was normalised to the expression in normal tissue. To differentiate between cases that overexpress a cyclin D protein from those that do not, we defined a value of 1.5-fold increased expression in the tumour, relative to normal tissue, above which a cyclin D protein was designated to be overexpressed. In order to account for the heterogeneity of the tissues, 15 out of 57 cases (nos. 15–29) were examined in duplicate, yielding identical results (data not shown).

Antibodies used in this study include anti-cyclin D1 (DCS-6), anti-cyclin D3 (DCS-22) (NeoMarkers, USA); anti-cyclin D2 (C-17) (Santa Cruz, USA); and actin (cp01) monoclonal mouse IgM (Oncogene, USA).

### mRNA analysis

Levels of carcinoembryonic antigen (CEA), mucin glycoprotein 3 (MUC3), cyclin D3 and *β*-actin were determined by reverse transcription–PCR (RT–PCR) of total RNA extracted from HT29 and LoVo39 cells treated with NaB. Total RNA extraction was performed with the RNeasy mini kit (Qiagen, Germany), with DNase (Qiagen, Germany) treatment to ensure removal of genomic DNA. The cDNA template was prepared using half of the RNA samples, using the first-strand synthesis kit (ABgene, UK). Primers for PCR amplification and product size were as follows: CEA, sense 5′-CAT GAT TGG AGT GCT GGT TG-3′, antisense 5′-ACC AAG CCC AGC TCA TTT T-3′, product size 270 bp; MUC3, sense 5′-AGT CCA CGT TGA CCA CCA CTG C-3′, antisense 5′-TGT TCA CAT CCT GGC TGG CG-3′, product size 406 bp; cyclin D3: sense 5′-CCC GAA AGG CGC AGT TGC AGC-3′, antisense 5′-GCC AGT GAT CCC TGC CAG CAG C-3′, product size 422 bp; and *β*-actin: sense 5′-ATG GAT GAT GAT ATC GCC GCG-3′, antisense 5′-CTA GAA GCA TTT GCG GTG GAC GAT GGA GGG GCC-3′, product size 238 bp. To visualise the amplification products, we performed PCR (Biometra, Germany). Cycling conditions were as follows: 95°C for 30 s, annealing for 30 s and 72°C for 60 s. The number of cycles was 35. Annealing temperatures for each product were as follows: 55.0°C for cyclin D3 primers, 56.3°C for CEA primers, 59.0°C for MUC3 primers and 60°C for *β*-actin primers. PCR products (10 *μ*l) were fractionated on 2% agarose gel; DNA was visualised by ethidium bromide staining and quantified by video densitometry (ImageMaster, Amersham, Germany).

### Cell cycle analysis

For cell cycle analysis, floating and adherent cells were fixed with 70% ethanol and stored at −20°C for at least 24 h. The fixed cells were collected by centrifugation and resuspended in 0.1% Triton X-100 and 30 *μ*g ml^−1^ RNase A type I-A (Sigma), at room temperature, for 40 min. Nuclear DNA was stained with 15 *μ*g propidium iodide in PBS solution and DNA content was measured by flow cytometry (FACSCalibur, Becton Dickenson, Mountain View, CA, USA). Cell proportions in sub-G0/G1, S and G2/M phases of the cell cycle were analysed using appropriate software. For each sample, 10 000 cells were scored.

### Alkaline phosphatase assay

Alkaline phosphatase (ALP) activity assay was performed following the biochemical technique described by Sabokbar *et al* (1994). The assay was performed by adding 50 *μ*l of the *p*-nitrophenyl phosphate substrate (Sigma, USA), final concentration 10 mM, dissolved in AMP-substrate buffer and 50 *μ*l of each test sample (cell lysate) to a 96-well microtitre plate. The cell lysate was prepared as follows: cells were washed with sterile PBS and resuspended in the same solution. Then, the number of viable cells was adjusted to 2 × 10^4^ and 5 × 10^4^ for HT29 cells and LoVo39 cells, respectively. The cell suspension was lysed by adding 1% (v v^−1^) Triton X-100 in PBS. The absorbance of the product (*p-*nitrophenyl phosphate) at 405 nm was measured at 37°C, using a Titertek Multiskan spectrophotometric plate reader (Molecular Devices, CA, USA). Alkaline phosphatase activity was measured at 1 min intervals, for 30 min. Alkaline phosphatase activity in NaB-treated cells was normalised to that in the control.

### Statistical analysis

The difference between the groups overexpressing the different D-type cyclins was evaluated by the two-tailed Fisher's exact test.

## RESULTS

### Expression of D-type cyclins during induction of proliferation

**Figure 1:**
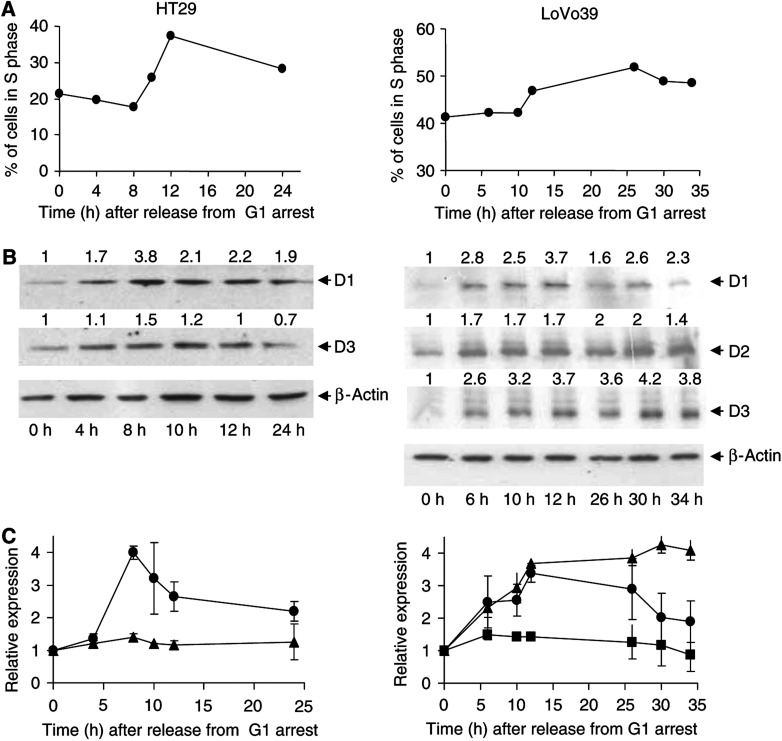
Expression of D-type cyclins during induction of proliferation. HT29 cells (left panels) and LoVo39 cells (right panels) were arrested in G1 phase of the cell cycle by serum starvation and then released. As a function of time following release, cell cycle phase distribution was analysed by flow cytometry (**A**), and D-type cyclin and *β*-actin protein expressions were analysed by Western blot analysis (**B**, **C**). In (**B**), representative Western blot analysis is presented. The locations of bands of the D-type cyclins and *β*-actin proteins, based on their molecular weight, are indicated by arrows. Numbers on top of each Western blot image represent the normalised expression of the various proteins (see Materials and Methods), relative to their expression at time of release from G1 arrest (0 h). Panel (**C**) displays the average relative expression of cyclin D1 (circles), D2 (squares) and D3 (triangles) as a function of time following release. The points and bars represent average and s.d. of three independent experiments.

Since D-type cyclins are believed to be cell cycle-promoting agents, we first studied their expression as a result of proliferation induction in HT29 and LoVo39 cell lines from colon carcinomas. In both cell lines, following release from serum starvation, an increase of cell population in S phase (Figure 1A) and a decrease in cell population in G1 phase (data not shown) were observed, indicating that the cells entered a cell division cycle. Population of cells in G2/M phase remained unchanged in both cell lines (data not shown). Induction of proliferation induced an increase in the expression of cyclin D1 in both cell lines (Figure 1B and C). The maximal increase in cyclin D1 expression, about four-fold relative to G1-arrested cells, was observed 8 h following release for HT29 cells and 12 h following release for LoVo39 cells, just at the beginning of the S phase of the cell cycle (Figure 1). The two cell lines exhibited differential expression pattern of cyclin D3 protein as a function of proliferation induction. In HT29 cells, the increase in cyclin D3 protein expression was moderate, only about 1.5-fold as compared to G1-arrested cells (Figure 1B and C, left panel). On the other hand, there was a four-fold increase in cyclin D3 protein expression following proliferation induction in LoVo39 cells (Figure 3B and C, right panel). Cyclin D2 protein expression in LoVo39 cells was also elevated as a function of induced proliferation. However, this increase was rather moderate (1.5-fold) and was considerably smaller as compared to the increase in expression of cyclin D1 and D3 proteins in the LoVo39 cell line (Figure 3B, right panel). These results indicate that although the three D-type cyclins participate in cell proliferation, their relative contribution differs.

### Expression of D-type cyclins during induction of differentiation

**Figure 2:**
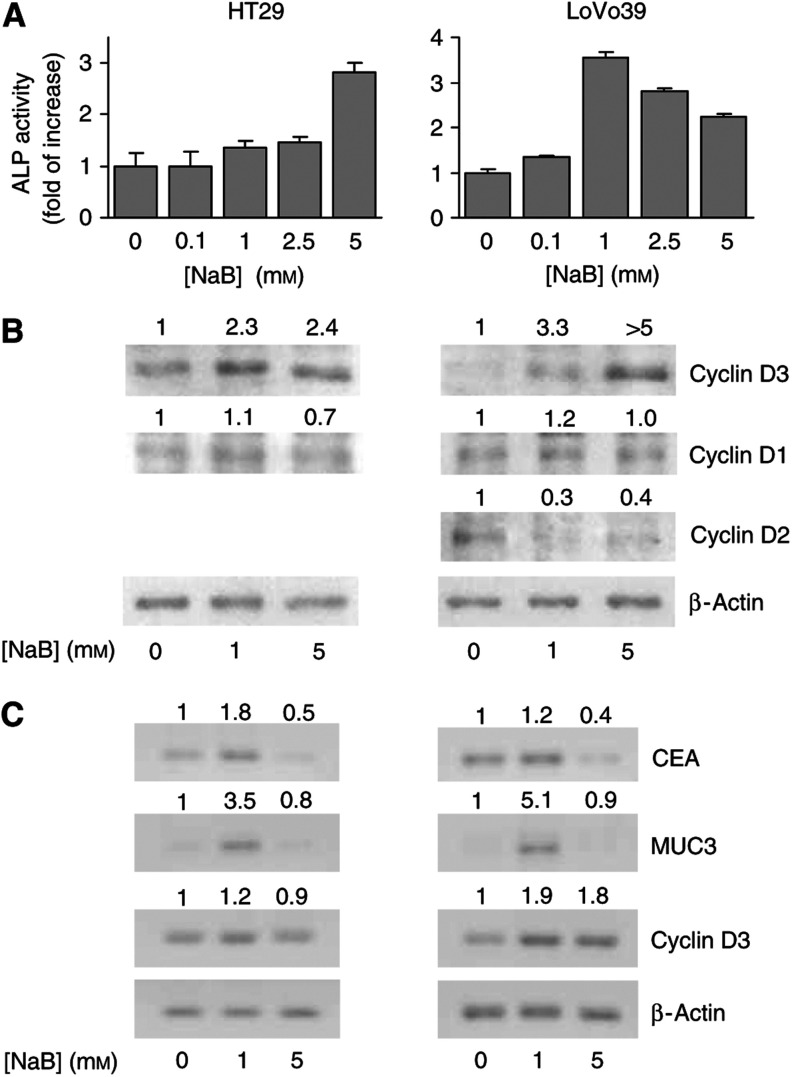
D-type cyclins expression during induction of differentiation by NaB. HT29 cells (left panels) and LoVo39 cells (right panels) were treated with NaB for 24 h. (**A**) Alkaline phosphatase (ALP) activity measured as a function of increased NaB concentrations (indicated on the abscissa). Columns and bars represent averages and s.d. of 3–4 experiments. (**B**) Cyclins D3, D2 and D1 and *β*-actin protein expression as a function of 24 h treatment with 1 and 5 mM NaB. Numbers at the top of the panels indicate protein levels of the different D-type cyclins, relative to *β*-actin expression and normalised to the relative expression in the control cells (0 mM NaB). Data are representative of 3–4 similar experiments. (**C**) mRNA expression of CEA, MUC3, cyclin D3 and *β*-actin (indicated on the right) as a function of 24 h treatment with 1 and 5 mM NaB. Numbers on top of the CEA, MUC3 and cyclin D3 panels represent mRNA expression relative to *β*-actin and normalised to relative expression of the corresponding gene in control cells (0 mM NaB). Data are representative of three experiments.

It has been previously shown that induction of differentiation is associated with elevation of cyclin D3 protein expression (Bartkova *et al*, 1998, 1999; Siavoshian *et al*, 2000). Therefore, in the present study, we examined the expression of D-type cyclins as a function of induced differentiation in HT29 and LoVo39 cell lines (Figure 2). Differentiation was induced by NaB, which is a well-known differentiation inducer of colonic epithelial cells (Kim *et al*, 1980). It has been demonstrated that NaB induces differentiation of colon-derived cell lines into mucus-secreting goblet cells that produce mucin glycoproteins and into absorptive enterocytes, which are characterised by elevated ALP activity (Wice *et al*, 1985; Schroy *et al*, 1994; Velcich *et al*, 1995; Gouyer *et al*, 2001). Induction of differentiation was followed by measurement of ALP activity in cell lysates (Colony and Neutra, 1983) and by RT–PCR detection of mRNA levels of two differentiation markers, CEA (Huitric *et al*, 1976; Velcich *et al*, 1995) and mucin 3 (MUC3) (Velcich *et al*, 1995; Gouyer *et al*, 2001; Gaudier *et al*, 2004). In both cell lines, 24 h treatment with NaB resulted in elevation of ALP activity (Figure 2A). In LoVo39 cells, ALP activity was induced by relatively low, 0.1 mM NaB concentration (Figure 2A, right panel). In these cells, the maximal increase (∼3.5-fold) in ALP activity was observed at 1 mM NaB. On the other hand, the increase in ALP activity of HT29 cells occurred at higher NaB concentrations and was more moderate, ∼2.5-fold at 5 mM NaB (Figure 2A, left panel). In addition, 1 mM NaB increased mRNA expression of CEA and MUC3 (Figure 2C), although in LoVo39 cells the increase in CEA mRNA was smaller compared to HT29 cells. We also observed that 5 days of treatment with 1 mM NaB induced the formation of what appears to be mucus-secreting vesicles in both cell lines (data not shown). These results indicate that NaB induces differentiation of HT29 and LoVo39 cell lines into both mucus-secreting cells and enterocytes.

Induction of differentiation by NaB was followed by a significant increase of cyclin D3 protein expression in both cell lines treated with 1 and 5 mM of NaB (Figure 2B). On the other hand, following NaB treatment, the expression of cyclin D1 remained unchanged while the expression of cyclin D2 decreased (Figure 2B). It has been suggested that during differentiation, elevation of cyclin D3 protein levels is a result of protein stabilisation (Cenciarelli *et al*, 1999; Siavoshian *et al*, 2000). Therefore, we examined cyclin D3 mRNA levels by RT–PCR in HT29 and LoVo39 cells treated with NaB (Figure 2C). In LoVo39 cells, the degree of elevation in cyclin D3 protein levels (Figure 2B, right panel) and in cyclin D3 mRNA levels (Figure 2C, right panels) was similar at both concentrations of NaB. In contrast, HT29 cells exhibited a different trend of elevation of cyclin D3 protein levels compared to mRNA levels. In these cells, which were treated with both 1 and 5 mM NaB, cyclin D3 protein expression was significantly higher than in untreated cells (Figure 2B, left panel), while only a moderate elevation (∼20%) in mRNA levels was observed in cells treated with 1 mM NaB. No change was observed following 5 mM NaB treatment (Figure 2C, left panel). These results suggest that in both cell lines, elevation in cyclin D3 protein levels following NaB treatment is at least in part transcriptionally regulated. In addition, these results clearly indicate that in HT29 cells, the differentiation-induced increase in cyclin D3 protein levels is also post-transcriptionally regulated.

### Expression of D-type cyclins in normal and cancer colon tissues

**Figure 3:**
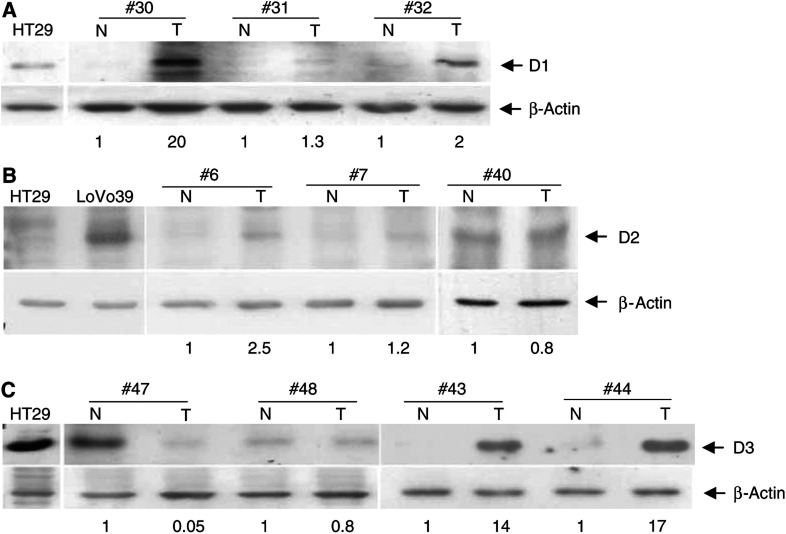
Expression of D-type cyclins in colorectal tumours. Western blot analysis of cyclin D1 (**A**), cyclin D2 (**B**) and cyclin D3 (**C**) protein expression in tumour (T) and adjacent normal (N) colon tissues. Representative results of several patients are shown (indicated by case number, on top). Protein extract from HT29 cells was used as a positive control for cyclin D1 and D3 protein expression and as a negative control for cyclin D2 protein expression. Protein extract from LoVo39 cells was used as a positive control for cyclin D2 expression. The locations of bands of the D-type cyclins and *β*-actin proteins, based on their molecular weight, are indicated by arrows. Numbers on the bottom of each Western blot image represent the normalised expression of D-type cyclin proteins in the tumour tissue (T), relative to their expression in the adjacent normal tissue (N).

Typical examples of expression of the three D-type cyclins proteins in tumour and adjacent normal colon tissue are shown in Figure 3. In cases 30 and 32, there was marked elevation of cyclin D1 protein levels, as compared to the adjacent normal tissue, while in case 31, there was no difference between the expression of the cyclin D1 protein between normal and cancer tissue (Figure 3A). We found that in agreement with previous reports (Arber *et al*, 1996, 1999; Sutter *et al*, 1997; Bukholm and Nesland, 2000; Kristt *et al*, 2000; Holland *et al*, 2001; Utsunomiya *et al*, 2001), cyclin D1 protein is overexpressed in 25 of 57 examined cases (44%) (Table 1). Cyclin D2 was also overexpressed in colon cancer tissue. For example, in case 6, cyclin D2 protein was overexpressed in tumour, as compared to the adjacent normal tissue. In cases 7 and 40, there is no difference between the expression of the cyclin D2 protein in normal and cancer tissue (Figure 3B). We found that in 30 of the 57 examined cases, cyclin D2 protein was overexpressed (53%) (Table 1). Typical examples of cyclin D3 expression in colon tissues are shown in Figure 3C. In cases 43 and 44, there was an elevation of cyclin D3 protein level in the tumour tissue. On the other hand, in case 48, there was similar expression of cyclin D3 protein in normal and cancer tissue. Unlike cyclins D1 and D2, which are overexpressed in cancerous tissue, in some cases cyclin D3 protein expression in the tumour was significantly lower (more than 10-fold) as compared to normal tissue (case 47 in Figure 3C). Cyclin D3 protein was overexpressed in 20 of 57 cases (35%) and underexpressed in nine of the cases (16%).

Tables 1 and 2 summarise the results regarding D-type cyclin protein expression. At least one D-type cyclin is overexpressed in 40 of 57 cases (70%), the majority (*n*=39, 68%) overexpressing either cyclin D1 or cyclin D2 (Table 1). This indicates that overexpression of cyclins D1 and D2 is related to colon carcinogenesis while overexpression of cyclin D3 is not. We also found that in 15 cases (26%), a single cyclin D protein was overexpressed in a given tumour, predominantly cyclin D2. In 15 cases (26%), two cyclin D proteins were overexpressed in the same tumour, the majority being pairs including cyclin D2. In 10 cases (17%), all three cyclin D proteins were overexpressed in the same tumour (Table 1).

We were unable to link between cyclin D protein expression and the grade of tumour differentiation, owing to the fact that the distribution of the tumour grades was highly disproportional and did not allow statistical analysis (see Materials and Methods). Therefore, more studies are needed to determine whether the overexpression of any of the D-type cyclins or underexpression of cyclin D3 can be related to the differentiation grade of the tumours.

We also examined the pattern of D-type cyclin overexpression in relation to the TNM stage of the tumours. Among the 57 cases, 38 were classified as TNM stages I and II, whereas 19 were classified as TNM stages III and IV in which metastases were found either in the lymph nodes (stage III) or at remote organs such as the liver or lungs (stage IV) (Table 2). There was no significant difference between the expressions of the three cyclins in TNM stage I and II tumours. On the other hand, the three D-type cyclins were overexpressed to a different degree in higher (III and IV) tumour stages. While the overexpression of cyclins D1 and D3 was similar, cyclin D2 overexpression (68%) was higher than that of cyclin D1 (37%, *P*=0.1) and cyclin D3 (32%, *P*=0.05) proteins (Table 2). The difference in overexpression between cyclin D1 and D2 did not reach the significance limit of 95%, being only 90% (*P*=0.1) (Table 2). However, our results suggest a 90% significant correlation between cyclin D2 protein overexpression and higher tumour stages.

## DISCUSSION

The main role of the three cyclin D family proteins is to promote cell cycle progression from G1 to S phase (Lukas *et al*, 1994). Indeed, we demonstrated that in two cell lines from colon carcinomas, protein expression of all three D-type cyclins increased as cells entered proliferation (Figure 1). Since most of the colon-derived cell lines do not express the cyclin D2 protein, this is the first study to indicate that in cell line from colon carcinoma, cyclin D2 protein expression is also elevated as a function of induced proliferation (Figure 1, right panels). In LoVo39 cells, the expression of cyclin D2 protein increased to a considerably smaller extent than cyclin D1 and D3 proteins (Figure 1, right panels). In HT29 cells, however, the increase in the expression of the cyclin D3 protein was significantly lower than that of the cyclin D1 protein (Figure 1, left panel). These results indicate that in different cell lines, D-type cyclins unequally contribute to cell proliferation.

Cyclin D1 overexpression has been reported to occur in 40–70% of colon tumours (Arber *et al*, 1996, 1999; Sutter *et al*, 1997; Bukholm and Nesland, 2000; Kristt *et al*, 2000; Holland *et al*, 2001; Utsunomiya *et al*, 2001). As such, 44% of cyclin D1 overexpression in the present study (Table 1) is in agreement with previous reports. Although overexpression and rearrangement of cyclin D1 gene are associated with tumour progression and/or poor prognosis in various tumour types, in colon cancer the link to prognosis is not clear (Bukholm and Nesland, 2000). We found that cyclin D1 overexpression is distributed equally among various TMN-stage tumours (Table 2), which indicates that in colon cancer, the overexpression of cyclin D1 is not related to the metastatic status of the tumour. This finding supports the idea that in the colon tissue, there is probably no link between the overexpression of cyclin D1 and prognosis.

In contrast to cyclin D1 protein, whose overexpression was distributed equally between the different TNM tumour stages, our data show a trend (*P*=0.1, 90% significance) that may point towards a positive relation between overexpression of cyclin D2 protein and higher TNM stage of the tumour (Table 2). This may suggest that the overexpression of cyclin D2, and not that of cyclins D1 and D3, is related to metastatic tumours. These results are in agreement with reports that overexpression of cyclin D2, but not of cyclin D1, in gastric cancer correlates with disease progression and poor prognosis (Takano *et al*, 2000). In addition, in flat and exophytic human colon adenomas, cyclin D2 was overexpressed in considerably larger proportions than cyclin D1 (Bartkova *et al*, 2001). Taken together, these findings seem to indicate that in the gastrointestinal tract, cyclin D2 acts as a proto-oncogene, and may have a predominant role in cancerous transformation. Moreover, in colon polyps, the overexpression of cyclin D2 was reported to be the most considerable aberration among several G1-phase regulators (Bartkova *et al*, 2001), indicating that the overproduction of cyclin D2 protein is an early event in neoplastic transformation of the colon. As such, and being positively correlated with the metastatic potential of the tumour (this study), cyclin D2 expression may serve as an early marker for colon tumorigenesis and for prediction of clinical prognosis of colon cancer. It has been suggested that hypermethylation of the CpG islands in the cyclin D2 promoter is related to the lack of cyclin D2 protein expression in breast cancer (Evron *et al*, 2001), prostate cancer (Padar *et al*, 2003) and gastric cancer (Yu *et al*, 2003), while hypomethylation of the CpG islands is related to the elevated expression of cyclin D2 in gastric cancer (Oshimo *et al*, 2003). It is possible that the overexpression of cyclin D2 in colon cancer is also related to promoter hypomethylation. Such cyclin D2 promoter hypomethylation may be an early event, which may explain the observed cyclin D2 overexpression in colon polyps (Bartkova *et al*, 2001) and possibly determine the metastatic potential of the colon tumour (Table 2).

Although the three D-type cyclins are believed to be functionally redundant in promoting cell cycle progression, they have been demonstrated to have other functions (Kato and Sherr, 1993). We have shown that in both HT29 and LoVo39 cell lines, cyclin D3 protein expression increased as a function of induced differentiation, while the expression of cyclins D1 and D2 remained unchanged or decreased (Figure 2B). This is in agreement with previous studies demonstrating a dual role for cyclin D3 protein in both proliferation and differentiation in several cell types (Bartkova *et al*, 1998, 1999; Siavoshian *et al*, 2000; Ito *et al*, 2001), including colon-derived cell lines (Siavoshian *et al*, 2000). In addition, it has been recently demonstrated that ectopic cyclin D3 expression induced differentiation-specific lamin reorganisation in C2C12 myoblasts (Mariappan and Parnaik, 2005), which suggests that ectopic cyclin D3 expression induces differentiation in these cells. At this point, it is not known whether ectopic cyclin D3 protein expression can also induce differentiation in colon-derived cells.

Although a growing amount of evidence has shown increased levels of cyclin D3 protein during differentiation in a number of cell lines and tissues, the mechanism by which increased cyclin D3 protein levels is related to differentiation is not clear. Data regarding the kinase activity of the cyclin D3–CDK4 complex during differentiation have revealed conflicting results. For example, it has been shown that during the differentiation of mouse 3T3-L1 adipocytes, protein levels of cyclin D3 and its association with CDK4 increased, while the association of p27 CDK inhibitor with this complex remained constant. Consequently, cyclin D3–CDK4 kinase activity remained high during differentiation in mature adipocytes (Phelps and Xiong, 1998). In addition, in the embryonal carcinoma cell line NTERA-2, cyclin E-dependent CDK2 activity, but not cyclin D3-dependent CDK4 activity, was suppressed as a result of hexamethylene-bisacetamide-induced differentiation (Baldassarre *et al*, 1999). On the other hand, during myogenesis, cyclin D3 protein levels and its association with CDK2 and CDK4 increased but cyclin D3-dependent kinase activity was markedly reduced (Kiess *et al*, 1995; Wang and Walsh, 1996). These findings suggest that cyclin D3 may have differentiation-related functions, which are CDK activity independent. It is clear that additional work is required to elucidate the mechanism by which increased cyclin D3 levels are related to differentiation of colon cells.

Recent studies have identified unique cyclin D3-associating proteins that are involved in protein transcription regulation but are not bound to cyclins D1 and D2 such as the mammalian transcription initiation factor 3 (Shen *et al*, 2004), 15-kDa cellular retinoic acid binding protein II and the nuclear retinoic acid receptor *α* (Despouy *et al*, 2003). It is possible that such proteins are involved in other cyclin D3 functions that differ from the cell cycle-promoting function. In addition, during skeletal muscle differentiation, activity of cell cycle-promoting CDK2 kinase is inhibited by interaction with the cyclin D3 and p27^kip1^ proteins (Chu and Lim, 2000), which could provide an alternative mechanism of cyclin D3 differentiation function. Since among the three D-type cyclins, only cyclin D3 protein is expressed in the differentiated regions of normal colon crypt (Bartkova *et al*, 2001), it is clear that elevated cyclin D3 protein levels observed in colon-derived cell lines (Siavoshian *et al*, 2000, and this study) reflect a unique role of this protein in differentiation of normal colon tissue. The findings that cyclin D3 was uniquely overexpressed in only one of the 57 cases (Table 1) and that in a subset of tumours cyclin D3 protein expression was reduced as compared to normal tissue (Figure 3C) support the notion that cyclin D3 plays an important role in the differentiation of colon epithelial cells but not in colon carcinogenesis.

To the best of our knowledge, this is the first report to characterise the expression of the three D-type cyclins in colon cancer tissue. Our data clearly indicate that overexpression of cyclin D1 and D2, but not D3, is related to cancerous transformation of the colon. Furthermore, the data suggest that cyclin D2 protein overexpression may be related to a higher stage of the tumour.

## Figures and Tables

**Table 1:** Cyclin D protein expression in colon tissue

	**Overexpression**	
**Cyclin D protein**	***n***	**%^a^**
No cyclin D overexpression	17	30
		
*At least one cyclin D protein in a tumour*		
D1, D2 or D3	40	70
D1 or D2	39	68
D1	25	44
D2	30	53
D3	20	35
		
*Single D-type cyclin overexpression in one tumour*		
D1	5	9
D2	9	16
D3	1	2
		
*Two D-type cyclins overexpression in the same tumour*		
D1 and D2	6	10
D1 and D3	4	7
D2 and D3	5	9
		
*Three D-type cyclins overexpression in the same tumour*		
D1, D2 and D3	10	17

a Calculated out of the 57 examined cases.

**Table 2:** Cyclin D protein expression relative to tumour staging

		**Overexpression**	
**TNM stage (*n*)**	**Cyclin D proteins**	***n***	**%^a^**
I and II (38)	D1	18	47
	D2	17	45
	D3	14	37
			
III and IV (19)	D1	7	37
	D2	13	68
	D3	6	32

a Calculated out of the 38 cases for stages I and II and out of the 19 cases for stages III and IV.
